# Prevalence and influencing factors of end-of-life symptoms among hospital decedents in South Korea: A cross-sectional secondary analysis

**DOI:** 10.1371/journal.pone.0357041

**Published:** 2026-08-25

**Authors:** Jooyoung Cheon, Eun Young Choi

**Affiliations:** 1 Department of Nursing Science, Sungshin Women’s University, Seoul, Republic of Korea; 2 Department of Nursing, Chung-Ang University, Seoul, Republic of Korea; University of Naples Federico II, ITALY

## Abstract

**Background:**

South Korea has the highest proportion of in-hospital deaths among OECD countries, yet population-based data on end-of-life symptom burden among hospital decedents remain scarce. This study examined end-of-life symptom prevalence in the last month of life and factors associated with each symptom among hospital decedents in South Korea.

**Methods:**

This cross-sectional secondary analysis used exit interview data from the Korean Longitudinal Study of Aging spanning 2006–2020. A total of 1,731 adults aged ≥45 years who died in hospital were included. Eleven proxy-reported symptoms were examined. Multivariable logistic regression with complex survey weights was used to identify factors associated with each symptom.

**Results:**

The mean age at death was 74.2 years (range: 47–103), and the mean symptom count was 4.76. The most prevalent symptoms were loss of appetite (66.5%), severe fatigue (59.4%), and depression (54.3%). Prolonged illness duration, functional dependence in ADL and IADL, and pain experience in the year before death were the factors most consistently associated with symptom occurrence across nearly all symptoms.

**Conclusion:**

These findings indicate that end-of-life symptom burden among hospitalized decedents in South Korea is high and consistently associated with prolonged illness duration, functional dependence, and pain. With only 4.51% of decedents having accessed hospice care before death, a critical gap exists between symptom burden and available palliative support. Early recognition of symptom indicators, integration of palliative care into hospital practice, and expanded access to hospice services are essential to improving the quality of dying.

## Introduction

As life expectancy increases and more people live with cancer and chronic illnesses, there is a growing societal demand for a dignified death or quality end-of-life care for older adults [1–3]. While many individuals express a preference to die at home [4,5], a significant proportion still pass away in healthcare facilities in South Korea [2]. The Organization for Economic Co-operation and Development (OECD) uses the proportion of deaths occurring in healthcare facilities as an indirect measure of the quality of end-of-life care [1]. South Korea recorded the highest rate, with 69.9% of deaths occurring in healthcare facilities, which is substantially higher than the 49.1% average across 36 OECD member countries during the same period [1]. By 2025, this rate had risen further to 75.7% [2]. This high proportion of in-hospital deaths is largely attributed to a lack of sufficient palliative care options outside of hospital settings and the expectation that hospitals can better manage uncontrolled symptoms associated with terminal illness [1,3,6,7].

For hospitalized patients at the end-of-life, effective symptom management is a top priority, as symptoms—reflecting changes in physical, psychosocial, sensory, or cognitive functioning—profoundly affect the ability to die with dignity and comfort [3,8,9]. Many of these symptoms are not adequately recognized or treated in the last months of life and tend to worsen as death approaches [10–13]. Inadequate symptom management can lead to functional decline, increased caregiver burden, and greater utilization of healthcare resources, such as higher rates of hospitalizations, readmissions, and emergency care [7,8,10,11]. Consequently, healthcare providers must be proficient in end-of-life symptom management and should take proactive steps to prevent and address common and frequent symptoms whenever possible [14].

Previous studies of end-of-life patients have shown that symptom burden increases significantly during the last months of life, influenced by demographic, socioeconomic, and disease-related factors [7,8,10,12,13,15–17]. Common symptoms experienced by these patients include pain, shortness of breath, fecal and urinary incontinence, sleep difficulties, loss of appetite, issues with nutrition/hydration, fatigue, lack of energy, drowsiness, delirium (or agitation), anxiety, and depression [7,8,10,13,17].

Factors influencing the frequency or severity of end-of-life symptoms include age, sex, socioeconomic status, employment, smoking habits, family support, type of illness, whether the patient is receiving active chemotherapy (for cancer patients), and impairments in activities of daily living (ADLs) and instrumental activities of daily living (IADLs) [12,13,15,16]. However, these factors vary depending on the type of symptom [15,16].

In South Korea, no studies have yet examined the prevalence of symptoms and the factors associated with each symptom among end-of-life patients who died in hospitals. The present study utilized data from the Korean Longitudinal Study of Aging (KLoSA), a nationwide panel survey that follows community-dwelling adults aged 45 and older across their entire life course—from middle age through death [18]. Uniquely among Korean panel surveys, KLoSA conducts post-mortem exit interviews with proxies (e.g., family members or close acquaintances) once a participant’s death is confirmed, enabling the collection of end-of-life data that would otherwise be inaccessible [18]. This approach is methodologically consistent with internationally recognized longitudinal aging studies, such as the U.S. Health and Retirement Study (HRS) [19] and the English Longitudinal Study of Ageing (ELSA) [20], which similarly employ proxy-reported post-mortem interviews to capture end-of-life experiences. Although proxy-reported data carry an inherent risk of recall bias, they remain an important and widely accepted source of information on the circumstances surrounding death, particularly when patient self-report is no longer possible [21,22].

Recognizing the need for research in this area is critical, as understanding the specific symptoms experienced in the last months of life can improve the quality of end-of-life care [10]. Identifying the prevalence and influencing factors of these symptoms is essential for assessing their burden and providing insights to healthcare providers and policy makers on enhancing end-of-life care. This study aimed to (1) determine the prevalence of end-of-life symptoms experienced in the last month of life among hospital decedents in South Korea, and (2) identify demographic, clinical, and care-related factors associated with these symptoms.

## Materials and methods

### Design

This study is a cross-sectional secondary analysis of data from the Korean Longitudinal Study of Aging (KLoSA), conducted by the Korea Labor Institute and the Korea Employment Information Service [18].

### Data source and participants

The KLoSA is an ongoing, nationwide longitudinal panel survey in South Korea that targets community-dwelling individuals aged 45 years and older. Participants are randomly selected using a multistage, stratified sampling method that accounts for housing type and geographic region, ensuring representation of the entire population of South Korea. Data collection occurs biennially through interviews, covering household background, socioeconomic status, and psychological and health-related characteristics.

The initial panel sample in 2006 comprised 10,254 middle-aged and older adults (born in 1961 or earlier). Survey retention rates were as follows: 86.6% in 2008, 81.7% in 2010, 80.1% in 2012, 80.4% in 2014, 79.6% in 2016, 78.8% in 2018, and 78.1% in 2020 [18]. Between 2008 and 2020, a total of 3,545 deaths were recorded among panel participants across the seven biennial survey waves. Exit interviews were conducted only in even-numbered years coinciding with core interview waves, with proxies such as family members or close relatives. Of the 3,545 deaths, proxy interviews were successfully completed for 2,727 decedents (76.9%), while interviews could not be conducted for the remaining 818 (23.1%) [18]. The proportion of successfully completed proxy interviews ranged from 71.8% to 82.6% across survey waves, with completion rates improving in more recent waves (80.7% in 2018; 82.6% in 2020). These exit interviews gathered information about the timing and circumstances surrounding the participant’s death, including symptoms experienced in the last month of life. All 2,727 decedents for whom proxy interviews had been successfully completed were initially included as the analytic population.

**Fig 1:**
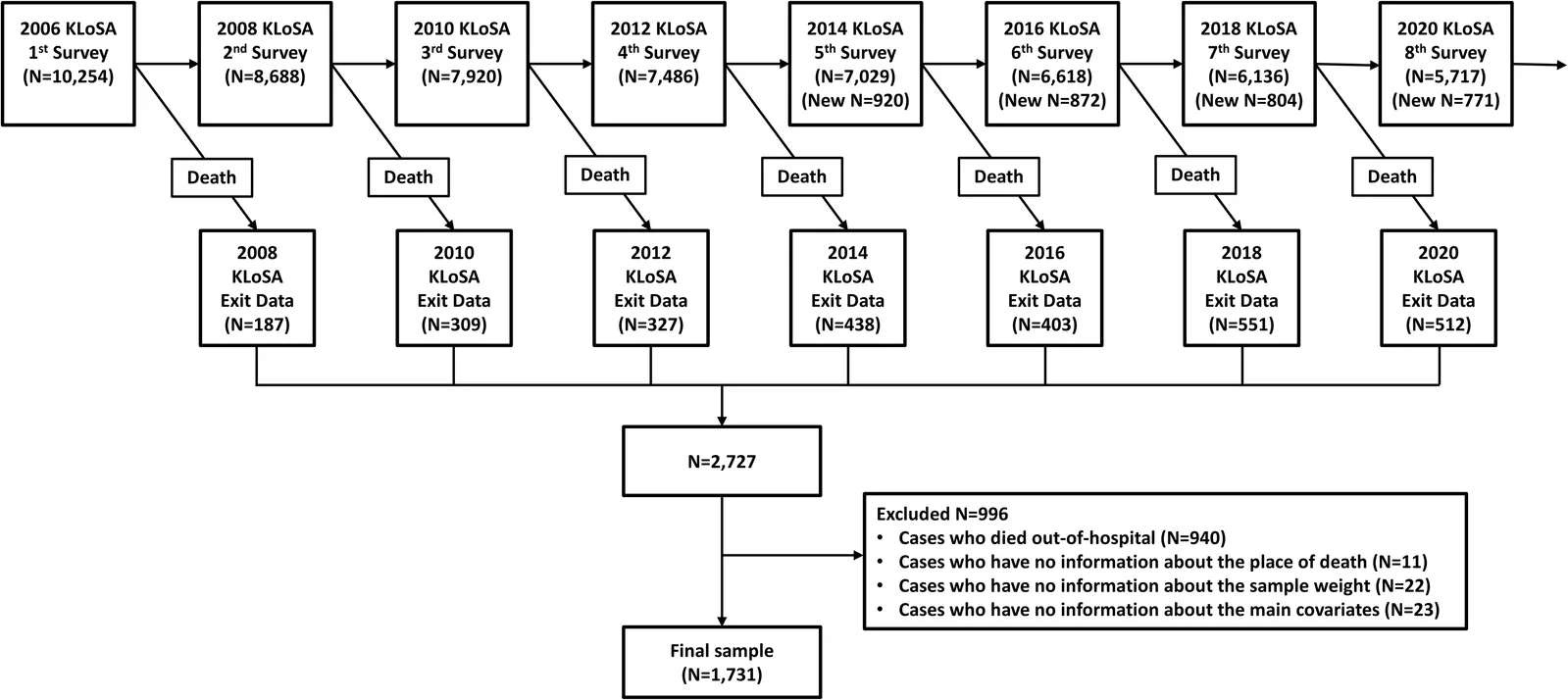
The sampling process in this study.

The target population of this study included individuals aged 45 years and older who died in hospitals between 2006 and 2020. Death in hospital was defined based on proxy-reported place of death. Specifically, in the KLoSA exit interview, proxies were asked, “Where did the deceased pass away?” with response options including home, hospital, nursing home or care facility, and other. Participants whose proxies indicated “hospital” were classified as having died in a hospital setting. Out of 2,727 deceased individuals identified, we excluded 940 who died outside of hospital settings, 11 with unspecified locations of death, 22 with missing sampling weight data, and 23 with missing information on key covariates. This resulted in a final sample of 1,731 participants who died in the hospital and were included in the analysis (Fig 1).

The authors accessed the KLoSA data for research purposes on 15 JAN 2024. The dataset provided to the authors was fully deidentified, and the authors did not have access to any information that could identify individual participants during or after data collection. Fig 1 provides an overview of the KLoSA survey process and the participant sampling methodology employed in this study. More detailed information about the KLoSA can be found on its official website (https://survey.keis.or.kr/eng/index.jsp).

### Measures

#### Outcomes.

Outcomes were defined as the symptoms experienced in the last month before death. 11 symptoms were assessed: dyspnea, persistent coughing, loss of appetite, frequent vomiting, difficulty urinating and defecating, discomfort in the extremities, severe fatigue, loss of consciousness, periodic mental confusion, depression, and difficulty controlling emotions [23]. Each symptom was measured using a single-item question. For example, to evaluate dyspnea, proxies were asked, “Did the deceased have trouble breathing a month before they died?” Responses to each item were originally recorded as either “yes,” “no,” or “don’t know.” For the purposes of this study, which focused on the presence or absence of each symptom, responses of “don’t know” were recoded as “no,” on the basis that an inability to confirm symptom occurrence was treated as absence of that symptom. Accordingly, each symptom was dichotomized as either “yes” (symptom present) or “no” (symptom absent).

#### Covariates.

This study also analyzed factors potentially influencing these symptoms, including demographic, socioeconomic, health status, and healthcare utilization factors. Demographic and socioeconomic variables included age at death, sex, educational level, marital status, religious affiliation, number of living children, and type of health insurance.

Physician-diagnosed comorbid chronic diseases included stroke, hypertension, diabetes, cancer, chronic lung disease (such as chronic bronchitis, emphysema, and chronic obstructive pulmonary disease), heart disease (including coronary artery disease, angina, and congestive heart failure), liver disease, dementia or memory disturbances, psychiatric disorders (such as depression), fractures or injuries from traffic accidents, and arthritis.

The experience of falls and pain in the year before death was assessed with a single question, with responses categorized as either “yes” or “no.” For falls, the question asked was, “Did the decedent have a fall between the time of the last survey and the time of death?” To evaluate pain, proxies were asked, “Did the deceased suffer from pain during the year before their death?”

ADLs during the three months preceding death were assessed across seven specific activities: dressing, washing one’s face and brushing hair/teeth, bathing or showering, eating, leaving the room after waking up, using the toilet, and controlling urination/defecation. IADLs during the same period were measured through ten tasks: grooming, performing routine housework, preparing meals, doing laundry, going out without transportation, going out with transportation, shopping, managing finances, making and receiving phone calls, and taking medications. Proxy responses for each item were scored as 0 (no help needed) or 1 (partial or complete help needed), with higher total scores indicating greater dependency. Total scores ranged from 0 to 7 for ADLs and from 0 to 10 for IADLs. No formal cut-off score for classifying dependency was established in the KLoSA, and both scores were therefore treated as continuous variables in the analyses.

The duration of illness before death was assessed using the question: “How long was the deceased ill before they passed away?” Responses were categorized into sudden death without prior illness, less than one day, one day to less than one week, one week to less than one month, one month to less than six months, six months to less than one year, one year to less than five years, and five years or more. For analysis of this study, these responses were re-categorized into five broader groups: (1) sudden death without prior illness, (2) more than 1 day but less than 6 months, (3) 6 months to less than 1 year, (4) 1 year to less than 5 years, and (5) 5 years or more.

Healthcare utilization factors included admission to a hospice ward or facility before death and the duration of the final hospitalization. In South Korea, hospice care may be provided either within a dedicated ward of a general hospital or in a separate, independent facility. Hospice admission was assessed using the question, “Since the baseline survey, how many times was the deceased admitted to a hospice ward or facility?” The duration of the most recent stay was additionally recorded for those with hospice admission experience (range: 1–730 days), while those with no hospice admission were assigned a length of stay of 0 days.

The duration of final hospitalization was assessed with the question, “How long was the deceased hospitalized before passing away?” Responses ranged from a minimum of 1 day to a maximum of 730 days. In this study, the final hospitalization lasting 31 days or more was considered hospitalization started more than 30 days prior to death for the purposes of analysis.

### Statistical analysis

All analyses were conducted using Stata 19 SE (StataCorp, College Station, TX, USA). In accordance with the KLoSA sampling method, the complex survey design was specified by declaring the primary sampling unit (PSU), stratum, and KLoSA-provided sampling weights.

Descriptive statistics were used to summarize the data, presenting unweighted numbers, weighted means and percentages along with their standard errors. To examine differences in the total number of symptoms across subgroups defined by categorical variables, survey-weighted linear regression was used. For variables with three or more categories, pairwise post-hoc comparisons were conducted with Bonferroni correction. To identify factors associated with the total number of symptoms, survey-weighted negative binomial regression was performed, given that the outcome exhibited overdispersion.

Multivariable logistic regression was conducted to investigate the associations between demographic, socioeconomic, health status, healthcare utilization factors and the type of symptom experienced in the last month of life. 11 separate models were fitted, one per symptom, with the same set of variables included in all models. Multicollinearity among covariates was assessed using variance inflation factors (VIF); all VIF values were within acceptable range. Because each of the 11 models addressed a distinct, prespecified symptom outcome rather than multiple comparisons within a single outcome, no correction for multiple testing was applied across models. As a sensitivity analysis, all 11 models were re-estimated excluding cases with “don’t know” responses for the corresponding symptom outcome. A two-tailed *p*-value of less than 0.05 was considered statistically significant.

### Ethical considerations

The KLoSA survey was conducted with state approval under Article 18 of the Statistics Act (approval number 33602), and all participants provided informed consent [18]. The KLoSA dataset is publicly accessible and can be downloaded free of charge from the official website (https://survey.keis.or.kr/kloee/kloee010.jsp). This study utilized a secondary analysis of a deidentified dataset from the KLoSA. As such, it was exempt from review by the Institutional Review Boards at the author’s institution (IRB no. SSWUIRB-2023–052).

## Results

### Participants’ characteristics

Between 2006 and 2020, a total of 1,731 participants who died in hospitals were included  (Table 1). The average age at death was 74.20 years (range 47–103), with slightly over half of the decedents (52.85%) being men. Approximately one-third (31.03%) had completed elementary school, the majority had spouses (68.79%), and most had no religious affiliation (69.10%). On average, decedents had 3.23 living children. Before their death, 90.08% of participants were covered by national health insurance.

**Table 1:** Participants’ characteristics (*N* = 1,731).

Variables	Unweighted Participant (*n*)	Weighted % or Mean^†^ (SE)	Weighted number of symptoms	
			Mean ± SD	*p*
Demographic and socioeconomic characteristics				
Age at death, y (range: 47–103)	1,731	74.20 (0.36)		
<60 ^a^	121	12.91 (1.16)	3.57 ± 2.76	<0.001
60–69 ^b^	266	21.98 (1.33)	4.12 ± 3.28	(a < c, d, e)
70–79 ^c^	531	28.36 (1.24)	5.18 ± 3.69	(b < c, d)
80–89 ^d^	610	28.11 (1.19)	5.32 ± 3.78	
≥90 ^e^	203	8.63 (0.69)	5.03 ± 3.89	
Sex				
Male	907	52.85 (1.29)	4.79 ± 3.72	0.776
Female	824	47.15 (1.29)	4.73 ± 3.58	
Educational level				
Uneducated ^a^	507	24.42 (1.16)	5.07 ± 3.65	0.016
Graduated from elementary school ^b^	563	31.03 (1.26)	4.95 ± 3.74	(d < a, b)
Graduated from middle school ^c^	243	15.10 (1.05)	4.98 ± 3.74	
Graduated from high school or higher ^d^	418	29.45 (1.46)	4.20 ± 3.41	
Marital status				
Having spouse	1,176	68.79 (1.38)	4.71 ± 3.72	0.397
No spouse	555	31.21 (1.38)	4.89 ± 3.51	
Religious affiliation				
Yes	531	30.90 (1.36)	4.68 ± 3.73	0.608
No	1,200	69.10 (1.36)	4.80 ± 3.62	
Number of living children (range: 0–9)	1,731	3.23 (0.05)		
Type of health insurance				
National Health Insurance	1,568	90.08 (0.92)	4.64 ± 3.65	<0.001
Medical Aid/long-term overdue/non-benefits	163	9.92 (0.92)	5.92 ± 3.51	
Health status				
Comorbid chronic diseases (range: 0–11)	1,731	4.00 (0.12)		
Stroke	679	35.75 (1.34)	5.09 ± 3.63	0.020
Hypertension	953	52.85 (1.46)	4.71 ± 3.63	0.571
Diabetes	762	41.56 (1.44)	5.01 ± 3.65	0.051
Cancer	815	45.63 (1.40)	5.64 ± 3.52	<0.001
Chronic lung disease	650	35.10 (1.33)	5.32 ± 3.52	<0.001
Heart disease	660	35.51 (1.34)	4.87 ± 3.55	0.464
Liver disease	534	28.99 (1.27)	5.03 ± 3.57	0.091
Dementia or memory disturbance	679	35.29 (1.32)	5.39 ± 3.63	<0.001
Psychiatric disease	533	28.09 (1.22)	4.91 ± 3.60	0.344
Fracture or injuries of traffic accident	528	28.04 (1.24)	4.80 ± 3.60	0.809
Arthritis	632	33.99 (1.34)	4.71 ± 3.60	0.726
Fall experience	244	13.10 (0.91)	6.49 ± 3.27	<0.001
Pain experienced in the year before death	630	35.99 (1.46)	6.94 ± 2.84	<0.001
ADL scores within 3 months before death (range: 0–7)	1,731	3.76 (0.10)		
IADL scores within 3 months before death (range: 0–10)	1,731	5.53 (0.14)		
Illness trajectory and healthcare utilization				
Duration of illness before death				
Sudden death without prior illness ^a^	283	20.64 (1.34)	0.94 ± 1.94	<0.001
More than 1 day but less than 6 months ^b^	546	29.11 (1.28)	4.65 ± 3.40	(a < b, c, d, e)
6 months to less than 1 year ^c^	256	14.10 (0.92)	6.17 ± 3.21	(b < c, d, e)
1 year to less than 5 years ^d^	455	25.52 (1.24)	6.27 ± 3.19	
5 years or more ^e^	191	10.63 (0.88)	7.02 ±3.02	
Admission to a hospice ward or facility before death	77	4.51 (0.64)	7.88 ± 2.77	<0.001
Length of stay in a hospice ward or facility before death (range: 1-730 days)	1,731	1.20 (0.47)		
Final hospitalization starting more than 30 days before death	312	17.2 (1.04)	7.04 ± 2.83	<0.001
Final hospitalization duration until death (range: 1-730 days)	1,731	33.58 (2.25)		

Abbreviations: ADL, activities of daily living; IADL, instrumental activities of daily living; SD, standard deviation; SE, standard error.

† Continuous variables were expressed as means and categorical variables as percentages.

The decedents suffered from an average of 4.00 common chronic illnesses, with the most prevalent conditions being hypertension (52.85%), cancer (45.63%), and diabetes (41.56%). Additionally, 13.10% had experienced at least one fall prior to death, and 35.99% had experienced pain during the year before death. In the three months before death, the average scores for ADLs and IADLs were 3.76 ± 0.10 and 5.53 ± 0.14, respectively. The duration of illness before death varied, with the most common period being between 1 day and less than 6 months (29.11%), followed by 1 year to less than 5 years (25.52%). Sudden death without prior illness occurred in 20.64% of cases.

A small proportion of decedents (4.51%) had been admitted to a hospice ward or facility before death, and 17.2% started their final hospitalization more than 30 days before death.

### The prevalence of symptoms experienced in the last month before death

**Fig 2:**
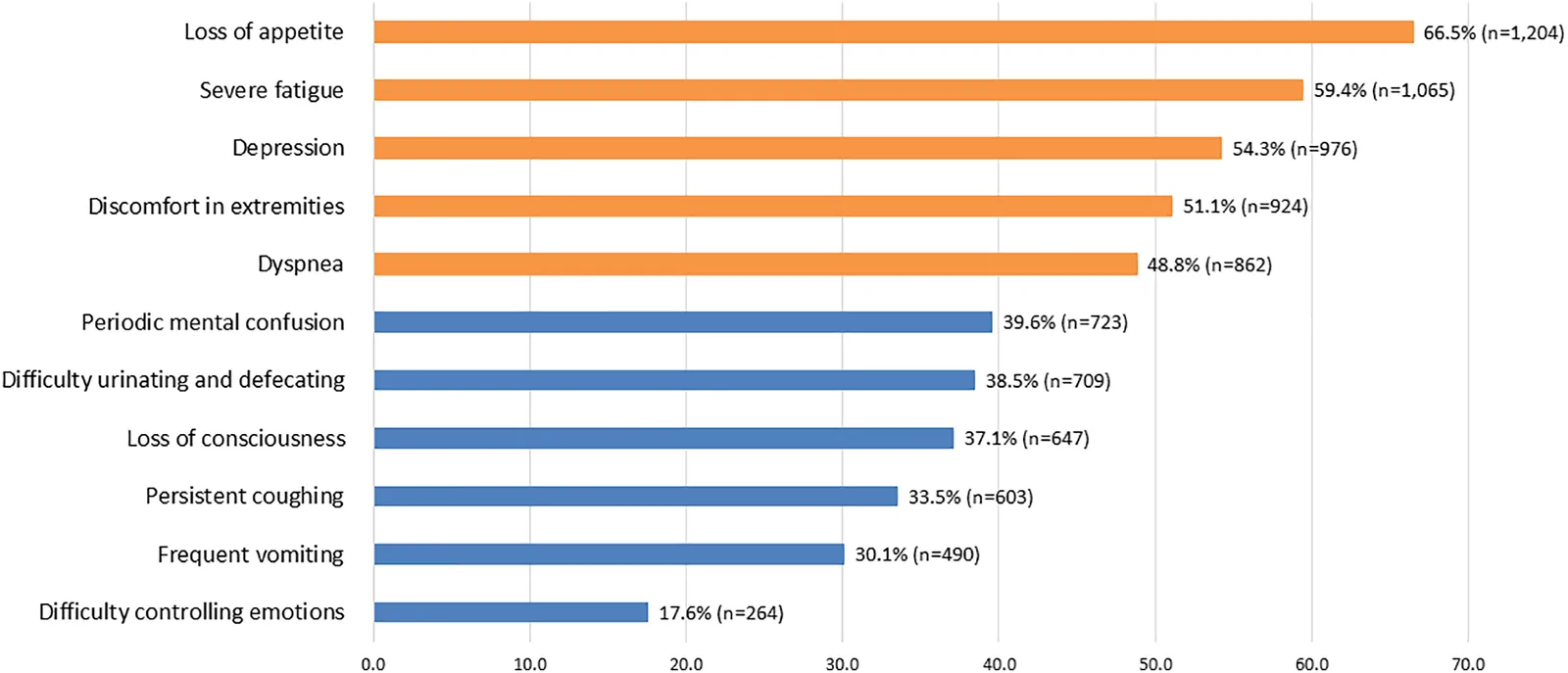
Prevalence of symptoms experienced in the last month before death (N = 1,731).

An analysis of the 15-year data revealed that the average number of symptoms experienced in the last month of life was 4.76 ± 0.12. The most common symptoms were loss of appetite (66.48%), severe fatigue (59.43%), and depression (54.25%), while the least frequent symptom was difficulty controlling emotions (17.58%) (Fig 2).

**Fig 3:**
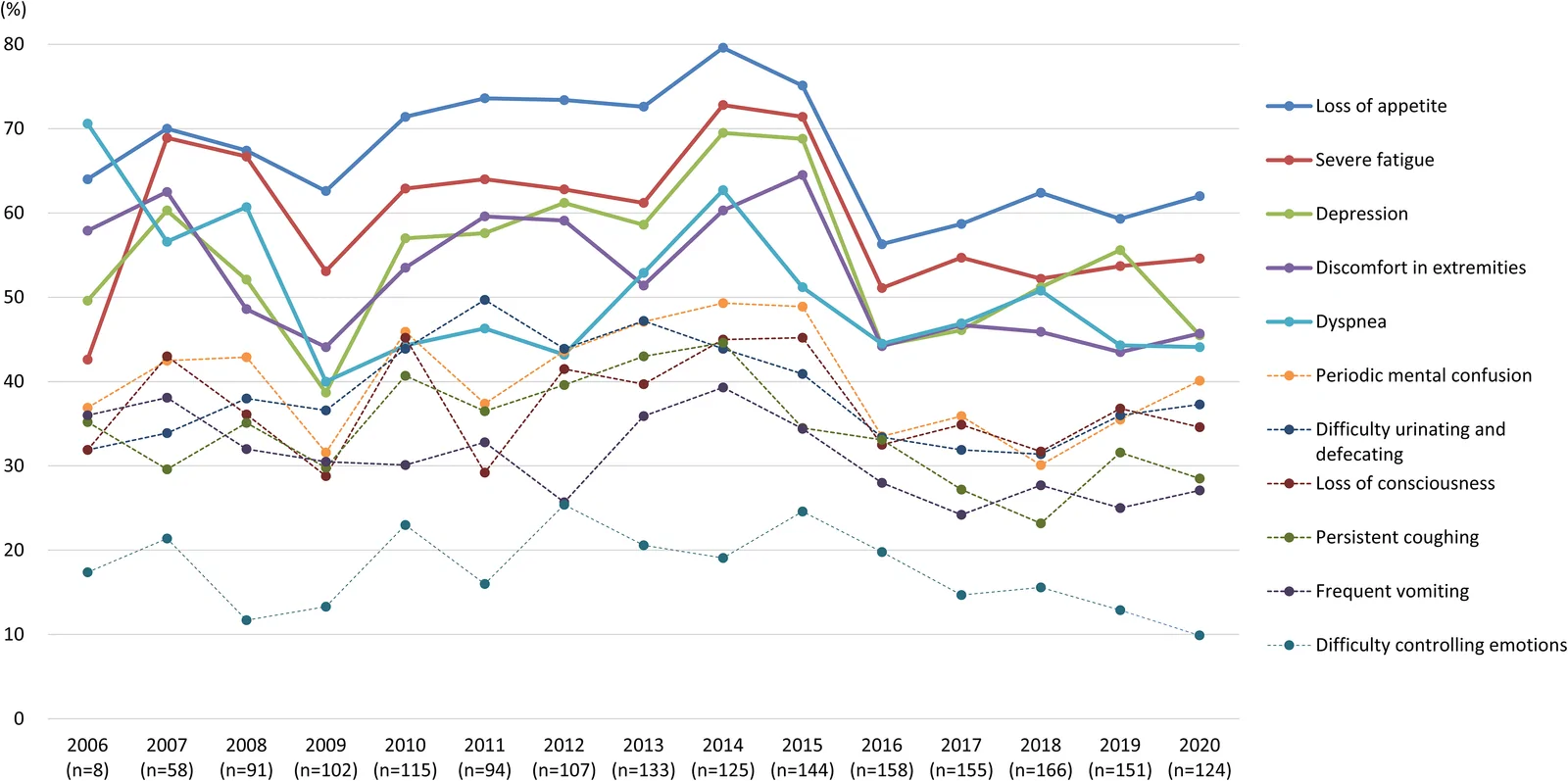
Trends in the prevalence of the 11 symptoms experienced in the last month of life (N = 1,731).

The prevalence rates of the 11 symptoms over the years were illustrated in Fig 3. Although slight variations were observed in the prevalence rates for all 11 symptoms across different years, their overall patterns remained consistent. Loss of appetite consistently showed the highest prevalence over the 15-year period, followed by severe fatigue, except in 2006 and 2019. Difficulty controlling emotions persistently exhibited the lowest prevalence throughout the entire study period.

The mean number of symptoms varied significantly across subgroups (S1 Table). Among demographic and socioeconomic characteristics, the mean number of symptoms was significantly higher in older age groups, those with lower educational attainment, and those covered by Medical Aid compared to national health insurance (all p < .05). Among health status variables, the mean number of symptoms was significantly higher in decedents with cancer, chronic lung disease, dementia, or stroke, and in those who had experienced falls or pain in the year before death, compared to their respective counterparts (all p < .05; S1 Table). With respect to illness trajectory and healthcare utilization, the mean number of symptoms was greater with longer illness duration, ranging from 0.94 ± 1.94 among those who died suddenly to 7.02 ± 3.02 among those ill for five or more years (p < .001). The mean number of symptoms was also significantly higher among decedents admitted to a hospice ward or facility (7.88 ± 2.77) and those whose final hospitalization began more than 30 days before death (7.04 ± 2.83; both p < .001). In the multivariable negative binomial regression, cancer, chronic lung disease, dementia, pain experience, ADL and IADL scores, illness duration, and hospice admission remained significant predictors of total symptom count (S2 Table).

### Factors influencing symptoms experienced in the last month before death

The results of the multivariable logistic regression for the three most prevalent symptoms (loss of appetite, severe fatigue, and depression) are presented in Table 2, with analyses for the other symptoms provided in S3 Table.

**Table 2:** Factors influencing major symptoms experienced in the last month before death (N = 1,731).

Variables	Odds ratio (95% CIs)		
	Loss of appetite (n = 1,204)	Severe fatigue (n = 1,065)	Depression (n = 976)
Health status			
Comorbid chronic diseases			
Stroke (yes)	0.761 (0.467-1.239)	0.897 (0.579-1.387)	0.750 (0.478-1.177)
Hypertension (yes)	1.001 (0.637-1.575)	1.116 (0.755-1.648)	1.005 (0.698-1.447)
Diabetes (yes)	0.901 (0.583-1.392)	1.035 (0.695-1.540)	1.252 (0.842-1.861)
Cancer (yes)	2.598 (1.596-4.231)***	1.898 (1.274-2.826)**	1.383 (0.908-2.109)
Chronic lung disease (yes)	2.064 (1.153-3.696)*	0.926 (0.596-1.439)	0.882 (0.526-1.479)
Heart disease (yes)	2.348 (1.441-3.827)**	1.569 (1.023-2.406)*	0.734 (0.460-1.172)
Liver disease (yes)	1.073 (0.447-2.574)	1.658 (0.764-3.599)	1.209 (0.490-2.980)
Dementia or memory disturbance (yes)	0.845 (0.468-1.523)	0.909 (0.573-1.440)	1.506 (0.960-2.363)
Psychiatric disease (yes)	1.073 (0.473-2.431)	1.090 (0.488-2.435)	2.897 (1.141-7.358)*
Fracture or injuries of traffic accident (yes)	0.281 (0.094-0.842)*	0.369 (0.124-1.095)	0.348 (0.113-1.072)
Arthritis (yes)	0.498 (0.298-0.830)**	0.641 (0.410-1.002)	0.546 (0.321-0.930)*
Fall experience (yes)	1.347 (0.702-2.588)	1.122 (0.712-1.768)	0.954 (0.629-1.445)
Pain experienced in the year before death (yes)	2.785 (1.778-4.361)***	2.394 (1.689-3.393)***	2.764 (1.990-3.839)***
ADL scores within 3 months before death	1.074 (1.002-1.151)*	1.092 (1.028-1.159)**	1.038 (0.973-1.107)
IADL scores within 3 months before death	1.175 (1.116-1.236)***	1.150 (1.101-1.202)***	1.126 (1.076-1.179)***
Illness trajectory and healthcare utilization			
Duration of illness before death			
Sudden death without prior illness	1 (ref)	1 (ref)	1 (ref)
More than 1 day but less than 6 months	7.399 (4.395-12.455)***	4.834 (2.919-8.008)***	5.747 (3.413-9.677)***
6 months to less than 1 year	6.749 (3.700-12.310)***	7.793 (4.25-14.293)***	8.684 (4.695-16.065)***
1 year to less than 5 years	6.185 (3.513-10.889)***	4.541 (2.623-7.862)***	7.439 (4.305-12.853)***
5 years or more	6.588 (3.320-13.072)***	6.666 (3.558-12.489)***	8.630 (4.556-16.348)***
Admission to a hospice ward or facility before death (yes)	1.792 (0.627-5.117)	1.029 (0.454-2.329)	2.749 (1.370-5.517)**
Final hospitalization starting more than 30 days before death (yes)	1.272 (0.774-2.089)	1.220 (0.820-1.816)	0.908 (0.615-1.341)

Note: The following demographic and socioeconomic variables were included in all models but are not shown in the table: age groups, sex, education level, marital status, religion, number of living children, and type of health insurance. Full results, including these variables, are presented in S3 Table.

Abbreviations: ADL, activities of daily living; CI, confidence interval; IADL, instrumental activities of daily living; ref, reference.

*p < 0.05; **p < 0.01; ***p < 0.001.

The primary factor associated with all symptoms was the duration of illness before death. IADL scores within three months before death were significantly associated with 10 symptoms, excluding persistent coughing. Additionally, pain experienced in the year before death was related to 9 symptoms, excluding difficulty urinating and defecating and persistent coughing.

The three most frequent symptoms experienced were influenced by several factors. Loss of appetite was significantly associated with cancer, chronic lung disease, heart disease, fracture or injuries of traffic accident, arthritis, ADL scores and IADL scores, pain experienced in the year before death, and the duration of illness before death. Severe fatigue was significantly associated with cancer, heart disease, ADL scores and IADL scores, pain experienced in the year before death, and the duration of illness before death. Depression was influenced by the number of living children, psychiatric disease, arthritis, IADL scores, pain experienced in the year before death, the duration of illness before death, and admission to a hospice ward or facility before death.

A sensitivity analysis excluding ‘don’t know’ responses yielded results largely consistent with the primary analysis, with minor discrepancies limited to borderline cases (S4 Table).

## Discussion

Among hospitalized decedents aged ≥ 45 years in South Korea, loss of appetite, severe fatigue, and depression were the most prevalent end-of-life symptoms in the last month of life, each affecting more than half of the study population. To our knowledge, this is the first population-based study to examine the prevalence of end-of-life symptoms and their associated factors specifically among hospital decedents in South Korea. Symptom prevalence patterns remained consistent across the 15-year study period, suggesting that the core symptom burden at the end of life is structurally embedded in terminal illness trajectories rather than fluctuating with changes in healthcare delivery. Prolonged illness duration, functional dependence in ADL and IADL, and pain experience in the year before death were the factors most consistently associated with symptom occurrence across nearly all symptoms examined.

In a study of advanced cancer patients who died in a specialist palliative care service, the mean number of symptoms was 5.8 per patient [5], comparable to the 4.76 symptoms observed in the present study across a broader, non-cancer-inclusive hospital population. Another study examining patients with serious illnesses in the six months before death reported that only 2.9 symptoms were appropriately screened and treated, with pain most effectively managed [7]. This pattern is further corroborated by Korean data: older adults who died in hospital settings experienced significantly higher odds of anorexia, depression, dyspnea, and confusion compared with those who died at home [24], and healthcare professionals in Korean tertiary hospitals have reported inadequate resources and structural barriers to effective end-of-life symptom management [25].

The predominance of loss of appetite, severe fatigue, and depression is consistent with findings from prior studies of terminally ill patients in other countries [12,13,15]. Loss of appetite is among the most consistently reported and distressing end-of-life symptoms, linked to disease progression, treatment side effects, and systemic inflammation [8,26,27], and its consistent predominance across all 15 years of this study reinforces its significance as a marker of illness progression [28]. Severe fatigue reflects the cumulative physiological burden of terminal illness and imposes substantial burden on both patients and their caregivers [11,29]. Depression, the third most prevalent symptom, is closely linked to the psychological burden of advanced illness and the anticipation of death [30,31], yet has received comparatively less attention in prior research [32]. The consistent co-occurrence of these symptoms across survey waves suggests that end-of-life symptoms are interconnected: as death approaches, physical symptoms tend to increase in prevalence [13,29] and mutually exacerbate one another—uncontrolled pain and depression amplify physical symptoms [13], while systemic inflammation contributes to the concurrent development of fatigue, pain, and anorexia [26,33].

Dyspnea ranked fifth in overall prevalence but was strongly associated with chronic lung disease and heart disease and was significantly more prevalent among those with prolonged final hospitalizations. Its relatively lower overall ranking may partly reflect proactive management in acute hospital settings; nevertheless, dyspnea remains a critical symptom in patients with cardiorespiratory conditions, consistent with evidence that breathlessness worsens near the end of life and is associated with increased hospital utilization [10,16]. The inverse associations between arthritis and multiple symptoms—including loss of appetite, depression, and discomfort in the extremities—are not readily explained by the present data. Possible hypotheses include adaptation to chronic pain [34], the anti-inflammatory effects of long-term NSAID use [35], or a disease trajectory distinct from that of patients with rapidly progressing malignancies [36]; however, these explanations remain speculative, and residual confounding by comorbidity pattern or disease severity cannot be excluded.

The strong associations between prolonged illness duration, functional dependence in ADL and IADL, pain, and overall symptom burden likely reflect shared underlying mechanisms rather than independent causal pathways. Given the cross-sectional study design, these associations should be interpreted as co-occurrence within a complex disease context rather than evidence of causal directionality. These factors are best understood as markers of advanced disease severity and progressive physiological deterioration. As terminal illness advances, the accumulation of systemic inflammation, metabolic dysregulation, and loss of functional reserve creates conditions conducive to multiple co-occurring symptoms [36,37]. In older patients approaching death, a reversal of conventional metabolic risk patterns—characterized by low body weight, low blood pressure, and declining physiological reserves—may reflect an underlying catabolic state driven by chronic illness and malnutrition, rather than the cardiometabolic risks typically seen in younger populations [38]. Functional dependence reflects not only physical impairment but also progressive loss of autonomy and engagement, contributing to depression and psychological distress [39]. Pain interacts bidirectionally with psychological symptoms: inadequately controlled pain exacerbates depression and emotional dysregulation, while depression amplifies the subjective experience of pain and other physical symptoms [40,41]. Effective pain management may therefore reduce the severity and frequency of co-occurring symptoms [40].

The hospital setting differs importantly from home or hospice care as a context for end-of-life symptom management. Hospitals prioritize acute disease management and life-sustaining treatments, and may lack the structural conditions for proactive, patient-centered symptom assessment that characterizes hospice and home-based palliative care [9]. Several factors specific to South Korea may compound this gap. Hospital admissions at the end of life in Korea frequently occur in response to acutely worsening symptoms rather than through early palliative care planning, meaning many patients arrive already experiencing substantial symptom burden [1,2]. Korean cultural norms—including family-based caregiving, reluctance to disclose a terminal prognosis to patients, and the expectation that hospitals can fully manage uncontrolled symptoms—may contribute to late palliative care referrals and continued aggressive treatment in the final weeks of life [2]. Additionally, the cultural significance of food in Korean caregiving may lead proxy respondents to overreport loss of appetite, while psychologically oriented symptoms such as depression and difficulty controlling emotions may be systematically underreported [21,46]. Symptoms such as loss of appetite and severe fatigue often persist despite hospital-based management, as they reflect the intrinsic biological trajectory of terminal illness [13,29]. One possible hypothesis is that the higher symptom burden observed among patients with prolonged final hospitalizations reflects delayed integration of palliative care principles rather than more effective symptom control; however, this interpretation could not be tested with the present data.

### Implications for clinical practice

The findings carry important implications for both clinical practice and health policy in South Korea. With 75.7% of all deaths occurring in healthcare facilities in 2025 [2], South Korea has the highest proportion among OECD countries [1]. The majority of dying patients are managed in acute hospital settings without adequate access to specialized palliative or hospice care. Although South Korea’s First Comprehensive Hospice and Palliative Care Plan (2019–2023) aimed to expand hospice services, utilization remains low and access is heavily concentrated among cancer patients [42,43]. The introduction of insurance-covered, home-based hospice care in September 2020 has shown promise in supporting home death among cancer patients [44], but the broader non-cancer population—who constitute a substantial proportion of hospital decedents—remains largely excluded from such services. Policies that incentivize early palliative care integration into hospital workflows, including systematic referral pathways to hospice services and routine symptom screening using validated tools, are therefore urgently needed.

At the clinical level, loss of appetite, severe fatigue, and depression—each consistently associated with prolonged illness duration, functional dependence, and pain—warrant priority attention in end-of-life symptom assessment, prompting timely palliative care involvement alongside continued disease-directed management [27,28]. Integrating symptom data with prognostic tools such as the Charlson Comorbidity Index can support patient stratification and resource allocation. The complexity and mutual reinforcement of end-of-life symptoms require a multidisciplinary approach involving palliative care teams, physicians, nurses, psychologists, and social workers [45]. For instance, patients experiencing concurrent loss of appetite and depression may benefit from care plans integrating nutritional support with psychological therapy, implemented through regularly updated clinical pathways [45]. Family involvement is central to effective end-of-life symptom management [9,16], and educational interventions that equip caregivers with skills to recognize and respond to symptom changes—including through family case conferencing [30]—are a priority for future research [9,46].

### Limitations

This study has several limitations. First, all symptom data were based on proxy reports from bereaved family members or close acquaintances. The exact date of each proxy interview was not recorded, so the actual interval between death and the proxy interview—which would more directly reflect recall delay—could not be calculated. As a reference, the interval between the participant’s last completed core interview and death was 2.80 ± 3.05 years (median: 1.60 years), though this reflects the timing of the participant’s last participation rather than the proxy interview itself. Nonetheless, because proxy interviews were necessarily conducted after the participant’s death, the possibility of recall bias cannot be excluded. Proxy responses may also reflect the proxy’s own emotional state rather than the patient’s actual symptom experiences. In particular, observable physical symptoms such as loss of appetite may be overreported, while psychologically oriented symptoms such as depression and difficulty controlling emotions are likely to be systematically underestimated [21,46]. Second, each symptom was assessed using a single binary item, which precludes evaluation of symptom severity, trajectory, or clinical significance. The yes/no format may also contribute to overestimation of symptom prevalence, as respondents may affirm the presence of a symptom without regard to its intensity or functional impact. Future studies should employ validated multidimensional tools such as the Edmonton Symptom Assessment Scale to capture both symptom presence and severity through patient self-report and proxy report [11,15]. Third, the cross-sectional nature of the exit interview data limits causal inference; associations between clinical factors and symptom occurrence should be interpreted as reflecting co-occurrence within a complex disease context rather than established causal relationships. Fourth, non-response bias cannot be excluded: of the 3,545 deaths recorded between 2008 and 2020, proxy interviews could not be completed for 818 decedents (23.1%), and the characteristics of these unobserved deaths could not be determined from the available data. Fifth, because no correction for multiple testing was applied across the 11 symptom models, some of the observed associations may reflect Type I error and should be interpreted with appropriate caution. Finally, the heterogeneous 15-year study period spans considerable changes in South Korea’s healthcare system and hospice infrastructure, which may have introduced unmeasured temporal confounding.

## Conclusion

Among hospitalized decedents aged ≥ 45 years in South Korea, loss of appetite, severe fatigue, and depression were the most frequent end-of-life symptoms over a 15-year period, each strongly associated with prolonged illness duration, functional dependence, and prior pain experience. These factors likely represent markers of advanced disease severity rather than independent causes, reflecting the biological trajectory of terminal illness. The persistently high symptom burden in a hospital setting—combined with very low hospice utilization—signals a critical gap between where people die in South Korea and where they can receive optimal end-of-life care. Early recognition of these symptom indicators, earlier integration of palliative care into hospital practice, and expanded access to hospice services are essential steps toward improving the quality of dying for hospitalized patients in South Korea.

## Supporting information

S1 Table General characteristics of the study sample (full distribution) (N = 1,731). (XLSX)

S2 Table Factors associated with total symptom count: results of survey-weighted negative binomial regression analysis (N = 1,731). (XLSX)

S3 Table Factors associated with 11 symptoms experienced in the last month before death (N = 1,731). (XLSX)

S4 Table Sensitivity analysis excluding “don’t know” responses for the 11 symptoms (N = 1,731). (XLSX)
