# Supplementary material for: Prevalence and influencing factors of end-of-life symptoms among hospital decedents in South Korea: A cross-sectional secondary analysis
Source: PLoS One. 2026 Aug 25;21(8):e0357041. doi: 10.1371/journal.pone.0357041 (PMC13505945; doi:10.1371/journal.pone.0357041)
Supplement: Supplementary file 1 [file pone.0357041.s006.docx]

**RESPONSE TO THE REVIEWERS**

Thank you very much for such thorough and scholarly feedback on this manuscript. The reviewer’s comments are invaluable in improving our manuscript. We present our responses to the comments made by the reviewer, as shown below:

**Editor**

| **Comments** | **Response** |
| --- | --- |
| 1) Structural aspects (abstract, explicit research questions, clarification of hospital vs. hospice care) | Thank you for this suggestion. Abstract condensed to ~250 words. Research aims explicitly stated at end of Introduction. Hospital admission and hospice admission clarified as distinct variables in the Methods and Discussion: in South Korea, hospice care may be provided within a dedicated ward of a general hospital or in a separate facility, and the two were assessed and analyzed separately in KLoSA. |
| 2) Important methodological issues (proxy-reported data, dichotomous symptom measures, potential misclassification and residual confounding). | Thank you for raising these concerns. Proxy-report methodology justified in the Introduction, citing HRS and ELSA. Limitations expanded to address: recall interval quantified (mean 2.80 ± 3.05 years; median 1.60 years), directional proxy bias (overreporting of physical, underreporting of psychological symptoms), and potential overestimation from binary response format. Place-of-death misclassification risk acknowledged in Methods and Limitations. Residual confounding addressed in Discussion. |
| 3) In the methods (definitions, handling of missing data, model specifications) | Thank you for this thorough review. Place of death defined as proxy-reported. "Don't know" responses recoded as "no" (conservative bias noted in Limitations). Exclusion counts specified in Figure 1 and Methods text. ADL/IADL scoring ranges (0–7; 0–10) and continuous treatment specified. Illness duration recategorization made consistent throughout. All analyses conducted in Stata 19 SE. |
| 4) Limited analytical depth (overly descriptive approach, lack of alternative models and subgroup analyses) | Thank you for this important critique. Supplementary survey-weighted negative binomial regression on total symptom count added (Table S2). Table 1 revised to include weighted mean symptom count per subgroup with p-values from survey-weighted linear regression; Bonferroni-corrected pairwise comparisons conducted for variables with ≥3 categories. Rationale for 11 separate logistic regression models explicitly justified in Methods. |
| 5) Improvements in data presentation and interpretation (e.g., counterintuitive ORs, insufficient discussion of the hospital setting) | Thank you for these suggestions. Table 1 reorganized into three thematic subgroups. Bar chart (Figure 2) added for overall symptom prevalence. Temporal trends across 15 years shown in Figure 3. Counterintuitive ORs (e.g., arthritis) explained in Discussion. Dyspnea ranking clarified. |
| 6) A stronger, more critical discussion and limitations section | Thank you for this recommendation. Discussion restructured into four sections: (1) main findings, (2) comparison with prior studies, (3) possible mechanisms, (4) clinical and policy implications. Limitations expanded: recall interval, proxy bias, non-response bias (23.1%), cross-sectional design, and temporal confounding. |

**Reviewer 1**

| **Comments** | **Response** |
| --- | --- |
| Although I praise the authors for this work and their effort, I doubt whether the new finding are newsworthy for an international public, also given the design with proxy responses. The major flaw is that the collected data are not only retrospective but also not provided by patients self but only by an interview with the next of kin after death of patients. In addition, the answers are dichotomous and not scaling, therefore yes or no, leading to overestimation perhaps. This all has a high risk of bias. This is however unavoidable given the database as the authors admit. | We thank the reviewer for this critique and fully acknowledge the limitations inherent to this study design.  Regarding international novelty, to our knowledge this is the first population-based study to examine end-of-life symptom prevalence and associated factors among hospital decedents in South Korea—the OECD country with the highest proportion of in-hospital deaths—with direct implications for palliative care policy in South Korea and comparable high-income Asian societies.  Regarding the proxy-report design, we have clarified in the Introduction that post-mortem proxy interviews are an internationally accepted methodology for capturing end-of-life experiences when patient self-report is not feasible, citing established longitudinal aging studies employing the same approach.  Regarding recall bias and binary measurement, we have expanded the Limitations section to quantify the recall interval (2.80 ± 3.05 years, median 1.60 years), acknowledge that proxy emotional state may lead to overreporting of physical symptoms and underreporting of psychological ones, and note that the yes/no response format may contribute to overestimation of symptom prevalence. We encourage readers to interpret all findings accordingly. |
| It is not known whether or not these symptoms were addressed during the hospital stay. To me it is surprising that so many symptoms exist in such a controlled environment with professional health care givers in a hospital. Could the authors comment on that? | We thank the reviewer for this insightful observation. The high symptom burden in a hospital setting may appear paradoxical, but several factors help explain it.  First, many of the most prevalent symptoms—such as loss of appetite, severe fatigue, and depression—are biologically intrinsic to advanced terminal illness and remain difficult to fully control regardless of care setting. Second, in South Korea, hospital admissions at the end of life frequently occur as emergency responses to acute deterioration rather than through early palliative care planning, meaning many patients arrive already carrying a substantial symptom burden. Third, hospitals in South Korea are structurally organized around curative care, and the integration of palliative principles remains limited. We have expanded the Discussion to address these points, and note that the higher symptom burden among patients with prolonged final hospitalizations further supports the view that extended hospital stays reflect delayed palliative care integration rather than effective symptom control. |
| The authors mention hospitalization but also hospice ward. Please explain, is there a difference according to the authors, is a hospice admission the same as an hospital admission or is a hospice a ward of a normal hospital or are there combinations? | We thank the reviewer for this important clarification request and have revised the Methods section accordingly.  In South Korea, hospice care can be provided either as a dedicated ward within a general hospital or as a separate, independent facility. Accordingly, hospice admission and hospital admission are not mutually exclusive—a patient counted as a hospital decedent may also have previously received hospice care. In the KLoSA exit interview, these two variables were assessed separately: hospice admission referred to any prior admission to a hospice ward or facility, while final hospitalization referred to the acute hospital stay immediately preceding death. Because the KLoSA does not specify whether the hospice ward was located within the same hospital where death occurred, both variables were retained as distinct in the analysis. |

**Reviewer 2**

| **Comments** | **Response** |
| --- | --- |
| **Abstract & Introduction** | Thank you for your feedback. |
| The abstract is overly long and includes background details better suited for the introduction. Condense it to 250–300 words, emphasizing the objective, key methods, major results, and main implications. | We have condensed the Abstract to approximately 250 words, removing background details now reserved for the Introduction. The revised Abstract focuses on the study objective, key methods, major results, and main implications, in line with the reviewer's recommendation. |
| The background repeats several ideas (functional decline, quality of dying, caregiver burden).  Streamline to highlight the knowledge gap—namely, the lack of studies examining symptom prevalence and associated factors among hospital decedents in Korea. | We thank the reviewer for this suggestion. We have streamlined the Introduction by removing repetitive statements and refocusing the narrative on the core knowledge gap: the absence of population-based studies examining end-of-life symptom prevalence and associated factors among hospital decedents in South Korea. We have also added a brief explanation of the methodological rationale for using proxy-reported data, noting that post-mortem proxy interviews are a widely accepted approach in large-scale end-of-life research when patient self-report is not feasible. |
| Clarify the research questions or hypotheses explicitly at the end of the introduction. For example:  “This study aimed to (1) determine the prevalence of end‑of‑life symptoms experienced in the last month of life among hospitalized decedents in South Korea, and (2) identify demographic, health, and care-related factors associated with these symptoms.” | The Introduction explicitly states the research aims as follows: “This study aimed to (1) determine the prevalence of end-of-life symptoms experienced in the last month of life among hospital decedents in South Korea, and (2) identify demographic, clinical, and care-related factors associated with these symptoms.”  We clarified the distinction between hospital and hospice care in both the Methods and Discussion sections. |
| **Methods** |  |
| The use of cross-sectional design on KLoSA exit interview data should be described more precisely. Indicate whether this secondary analysis included all proxy respondents or only those with complete symptom data. | We thank the reviewer for this methodological comment. We have revised the Methods section to clarify that this study employed a cross-sectional secondary analysis of KLoSA exit interview data. We have also explicitly stated that all 2,727 decedents for whom proxy interviews were successfully completed were initially included as the analytic population, and that the final analytic sample of 1,731 was reached through sequential exclusion of non-hospital decedents, cases with unspecified place of death, and those with missing sampling weights or key covariates. |
| Clarify how “death in hospital” was defined—e.g., based on proxy report or KLoSA administrative linkage—and discuss potential misclassification. | We thank the reviewer for this important methodological point. We have revised the Methods section to clarify that place of death was defined based on proxy report in the KLoSA exit interview, not administrative linkage or death certificates. We acknowledge that this approach carries a risk of misclassification—for example, proxies may have been uncertain about the distinction between a hospital and a care facility—and have noted this explicitly in both the Methods and Limitations sections. As KLoSA does not include administrative linkage for place of death, proxy-reported classification represents the most feasible approach available for this study. |
| The exclusion process shown in Fig. 1 must specify how many participants were removed for missing covariates or weighting data. | We thank the reviewer for this comment. We have revised Figure 1 and the corresponding Methods text to explicitly report the number of participants excluded at each step: 940 excluded for non-hospital place of death, 11 for unspecified location, 22 for missing sampling weight data, and 23 for missing information on key covariates, resulting in a final analytic sample of 1,731. |
| List each symptom exactly as coded in KLoSA, specifying the response categories and how missing or “don’t know” responses were handled. | We thank the reviewer for this point. We have revised the Methods section to specify that each symptom was assessed using a single-item question with three original response options ("yes," "no," "don't know"), and that "don't know" responses were recoded as "no" on the basis that inability to confirm symptom presence was treated as absence. We acknowledge that this approach may introduce conservative bias, as some symptoms coded as "no" may have been present but unknown to the proxy, and have noted this in the Limitations section. |
| The ADL and IADL scores should include the scoring range and the threshold, if any, for “dependency.” | We thank the reviewer for this clarification request. We have revised the Methods section to specify the scoring range (ADL: 0–7; IADL: 0–10) and to clarify that although the original instruments use a 3-point scale, KLoSA responses were recoded as binary scores (0 = no help needed; 1 = partial or complete help needed) per item. We have also noted that no formal cut-off score for "dependency" was established in the original instrument development studies, and that ADL and IADL scores were therefore treated as continuous variables in the analyses. |
| Clarify how the variable duration of illness before death was re‑categorized for regression analysis—currently presented inconsistently (lines 161–164 vs. 263 table). | We thank the reviewer for identifying this inconsistency. We have revised the Methods section to explicitly specify the five re-categorized groups used in the regression analysis: (1) sudden death without prior illness, (2) more than 1 day but less than 6 months, (3) 6 months to less than 1 year, (4) 1 year to less than 5 years, and (5) 5 years or more. This categorization is now consistent with the categories presented in the tables throughout the manuscript. |
| More justification is needed for using 11 separate logistic regressions rather than a multivariate or count‑based model (e.g., Poisson or negative binomial) for symptom number. | We thank the reviewer for this important methodological comment. The primary aim of this study was to identify factors associated with each individual symptom, as the clinical utility of findings depends on understanding which specific symptoms are predicted by which factors. A single count-based model would estimate the effect of each covariate on total symptom burden, obscuring these symptom-specific associations and limiting clinical applicability. Prior evidence also suggests that predictors of end-of-life symptoms vary substantially by symptom type [Battat, 2023; Conen, 2021], further supporting the use of symptom-specific models. Eleven separate binary logistic regression models were therefore fitted, one per symptom, to characterize distinct predictor profiles for each outcome.  To address the reviewer's concern regarding the total symptom count as an outcome, we have added a supplementary analysis using negative binomial regression, given that the total symptom count exhibited overdispersion. Results are presented in Table S2 and are consistent with the primary findings, with illness duration, IADL dependence, and pain remaining the strongest predictors of overall symptom burden. |
| Indicate which confounders were included in each model and whether multicollinearity was tested. | We thank the reviewer for this point. All 11 logistic regression models included the same set of demographic and socioeconomic variables as covariates: age group, sex, educational level, marital status, religious affiliation, number of living children, and type of health insurance. These variables were selected a priori based on theoretical relevance and prior literature. Multicollinearity among covariates was assessed using variance inflation factors (VIF); all VIF values were within acceptable range, indicating no problematic collinearity. The Methods section has been revised accordingly. |
| Specify how sampling weights were applied in SPSS (complex sample module, strata, cluster). | We thank the reviewer for this clarification request. We have revised the Methods section to specify that all analyses were conducted using Stata 19 SE (StataCorp, College Station, TX, USA), with the complex survey design declared by specifying the primary sampling unit (PSU), stratum, and KLoSA-provided sampling weights. Stata was adopted as the analytical platform to accommodate the supplementary negative binomial regression analysis added in response to the reviewers' comments, ensuring consistency across all analyses. |
| Include the total number of observations used in each regression model to assess sample attrition. | We thank the reviewer for this suggestion. The total number of observations used in each regression model is presented in the header of Tables 2 and Table S3 (e.g., Loss of appetite, n = 1,204), confirming that all models were fitted on the analytic sample with no additional case-wise exclusion at the regression stage. |
| **Results** |  |
| Tables 1–3 are informative but dense. Simplify by grouping variables thematically or relocating supplementary details to an appendix. | We thank the reviewer for this suggestion. We have addressed this concern in the following ways. First, Table 1 has been reorganized into three thematic subgroups—demographic and socioeconomic characteristics, health status, and illness trajectory and healthcare utilization—to improve readability. For health status variables measured as yes/no (e.g., comorbidities, fall experience, pain experience, hospice admission), only the "yes" group is presented, reducing the number of rows. Categorical variables with multiple response options (e.g., age group, sex, educational level, marital status) retain all categories. Second, the prevalence data previously presented in table format have been converted to a bar chart (Figure 2), providing a more accessible visual summary. Third, Table 2 has been retained in its current form, as it presents the primary regression results for the three most prevalent symptoms and is essential for interpreting the main findings. Results for the remaining eight symptoms are provided in Table S3. |
| Provide unweighted n and weighted % or mean consistently. | We thank the reviewer for this comment. Table 1 has been revised to present both unweighted n and weighted % or mean (SE) consistently for all variables, alongside the weighted mean number of symptoms (Mean ± SD) and corresponding p-values for subgroup comparisons. |
| Consider a separate figure with stacked bars showing the top five symptoms to visualize overall burden more clearly than the current textual description. | We thank the reviewer for this suggestion. We have added a bar chart (Figure 2) displaying the weighted prevalence of all 11 symptoms in descending order, with the top five symptoms visually distinguished in orange. We opted for individual bars rather than stacked bars, as the 11 symptoms are non-mutually exclusive binary outcomes—a single decedent may have experienced multiple symptoms simultaneously—meaning the prevalences do not sum to 100% and are therefore not suitable for a stacked format. In addition, we have retained the line graph (Figure 3) to illustrate temporal trends in symptom prevalence across the 15-year study period (2006–2020). Although slight year-to-year variation is observed, the overall rank order of symptoms remained consistent, with loss of appetite persistently showing the highest prevalence and difficulty controlling emotions the lowest throughout the entire study period. |
| The mean number of 4.76 ± 0.12 symptoms is reported, but no measure of distribution or comparison by subgroups (e.g., age, sex, illness duration) is offered. Include analyses showing how symptom burden varies across key characteristics. | We thank the reviewer for this suggestion. Table 1 has been revised to include the weighted mean number of symptoms (Mean ± SD) for each subgroup, along with p-values from survey-weighted linear regression comparing the mean number of symptoms across subgroups defined by categorical variables. For categorical variables with three or more categories (e.g., age group, educational level, illness duration), pairwise post-hoc comparisons were conducted with Bonferroni correction applied within each variable to control for multiple comparisons across categories. This correction was applied to the subgroup mean comparisons in Table 1 and does not extend across the 11 separate logistic regression models, which were each fitted for a distinct symptom outcome. In addition, survey-weighted negative binomial regression was performed to identify factors independently associated with the total number of symptoms, given that the outcome exhibited overdispersion; results are presented in S2 Table. These analyses are described in the Methods section, and results are reported in the Results section. |
| Some odds ratios appear counterintuitive (e.g., arthritis inversely associated with loss of appetite and depression). Discuss potential confounding, measurement issues, or clinical explanations. | We thank the reviewer for this observation. We have expanded the Discussion to address the inverse associations between arthritis and loss of appetite, depression, and discomfort in the extremities. Plausible explanations include adaptation to chronic pain, the anti-inflammatory effects of long-term NSAID use, and a disease trajectory distinct from that of patients with rapidly progressing malignancies. We also acknowledge that residual confounding by comorbidity pattern or disease severity cannot be excluded. |
| The statement that dyspnea was not a “major symptom” contradicts its 48.8 % prevalence—clarify your comparison criteria. | We thank the reviewer for identifying this ambiguity. We have revised the Discussion to clarify that dyspnea ranked fifth in overall prevalence (48.8%), making it one of the most frequently reported symptoms in this cohort. |
| **Discussion** |  |
| Currently too long and largely descriptive. Organize around (1) main findings, (2) comparison with prior studies, (3) possible mechanisms, and (4) clinical implications. | We thank the reviewer for this suggestion. We have substantially reorganized the Discussion around four sections: (1) main findings, summarizing the overall symptom burden and most consistently associated factors; (2) comparison with prior studies, contextualizing our findings against international evidence on symptom prevalence and treatment adequacy; (3) possible mechanisms, addressing why prolonged illness duration, functional dependence, and pain are so consistently linked to symptom burden, with reference to shared biological pathways including systemic inflammation and catabolic processes; and (4) clinical and policy implications, focusing on the structural and cultural factors specific to the Korean hospital context and the need for earlier palliative care integration. Redundant and purely descriptive passages have been removed. |
| Avoid repeating numerical results; interpret instead what these indicate about end‑of‑life care quality or system gaps. | We thank the reviewer for this suggestion. We have revised the Discussion to minimize repetition of numerical results, focusing instead on interpreting what the observed patterns indicate about end-of-life care quality and system gaps in South Korea. Numerical comparisons are retained only where they serve a direct interpretive purpose, such as contextualizing the overall symptom burden against international benchmarks. |
| The discussion should address why the duration of illness, ADL/IADL dependence, and pain are so strongly linked with most symptoms. Are these causal, or proxies for disease severity? | We thank the reviewer for this important question. We have revised the Discussion to clarify that prolonged illness duration, ADL/IADL dependence, and pain are best understood as markers of advanced disease severity rather than independent causes of individual symptoms. Given the cross-sectional design, the observed associations reflect co-occurrence rather than causal directionality, likely mediated by shared biological pathways including systemic inflammation, metabolic dysregulation, and progressive loss of functional reserve. |
| Include a more explicit reflection on the hospital setting—why symptom management may differ from home or hospice deaths | We thank the reviewer for this suggestion. We have expanded the Discussion to address why symptom burden may remain high in hospital settings despite professional care. Hospitals are structurally oriented toward curative and life-sustaining treatment rather than comfort-focused care, and in South Korea, terminal hospitalizations typically occur as emergency responses to acute deterioration rather than through early palliative planning. Cultural norms around prognosis disclosure and family-centered caregiving further contribute to late palliative care referrals. These factors collectively explain why hospital care may fail to adequately address end-of-life symptom burden, in contrast to hospice or home-based settings where symptom management is the primary goal. |
| Expand on policy implications in Korea’s current hospice‑care context rather than only recommending “multidisciplinary care.” See and discuss:  Depression:  1) Cacciatore F et al.. Effect of Sacubitril-Valsartan in reducing depression in patients with advanced heart failure. J Affect Disord. 2020 Jul 1;272:132-137.  Frailty and heart failure:  2) Testa G et al. Multidimensional frailty evaluation in elderly outpatients with chronic heart failure: A prospective study. Eur J Prev Cardiol. 2019 Jul;26(10):1115-1117.  Catabolic syndrome:  3 Curcio F et al. The reverse metabolic syndrome in the elderly: Is it a "catabolic" syndrome? Aging Clin Exp Res. 2018 Jun;30(6):547-554. | We thank the reviewer for these suggestions. We have expanded the policy implications section beyond generic recommendations for multidisciplinary care to address South Korea's specific hospice and palliative care context, including the structural gap between high in-hospital death rates and low hospice utilization, the exclusion of non-cancer patients from hospice services, and the need for earlier palliative care integration into hospital workflows. We have also incorporated discussion of the catabolic syndrome concept (Curcio et al., 2018) to provide a biological basis for the co-occurrence of weight loss, anorexia, and fatigue in older patients approaching death, linking mechanistic understanding to the rationale for targeted symptom interventions. We appreciate the suggestion to cite Cacciatore et al. (2020) and Testa et al. (2019); however, as our study population is not limited to heart failure patients and these references fall outside the primary scope of our findings, we have opted not to include them in the current revision. |
| Quantify the potential recall bias (delay between death and exit interview). | We thank the reviewer for this suggestion. We have quantified the potential recall interval in the Limitations section. The interval between the last core interview and the participant's death was 2.80 ± 3.05 years (median: 1.60 years), suggesting that proxy interviews may have been conducted a considerable time after death. We acknowledge that longer intervals may increase the risk of inaccurate or emotionally distorted recall of the decedent's last month of life. |
| Discuss non‑response bias and whether excluded cases differed systematically. | We thank the reviewer for this suggestion. We have added discussion of non-response bias in the Limitations section, noting that proxy interviews could not be completed for 23.1% of decedents. As the characteristics of these unobserved cases could not be determined from the available data, we cannot confirm whether excluded decedents differed systematically from those included, and acknowledge that this represents a potential source of selection bias. |
| Note the limited ability to infer causality due to the cross‑sectional design. | We thank the reviewer for this point. We have explicitly noted in the Limitations section that the cross-sectional nature of the exit interview data precludes causal inference, and that all associations should be interpreted as reflecting co-occurrence within a complex disease context rather than established causal relationships. |
| Mention that proxy perceptions may systematically underestimate psychological symptoms (depression, emotion control). | We thank the reviewer for this point. We have noted in the Limitations section that proxy respondents may systematically underestimate psychologically oriented symptoms such as depression and difficulty controlling emotions, as these are less observable and less culturally salient as caregiving concerns compared with physical symptoms such as loss of appetite. |
| **Conclusion** |  |
| Condense to one clear paragraph emphasizing what was discovered and how it advances practice.  Avoid generic statements like “this study provides foundational data.” Instead:  “Among hospitalized decedents aged ≥45 years in Korea, loss of appetite, severe fatigue, and depression were the most frequent end‑of‑life symptoms, each strongly associated with prolonged illness and higher dependency. Early recognition of these indicators may improve the quality of dying through targeted palliative interventions.” | We have condensed the Conclusion to a single focused paragraph that emphasizes the key findings and their practical implications, avoiding generic statements. The revised Conclusion highlights the most prevalent symptoms and their associated factors, interprets these as markers of disease severity, and directly connects the findings to actionable recommendations for palliative care practice and policy in South Korea. |
