# Supplementary material for: Prevalence and influencing factors of end-of-life symptoms among hospital decedents in South Korea: A cross-sectional secondary analysis
Source: PLoS One. 2026 Aug 25;21(8):e0357041. doi: 10.1371/journal.pone.0357041 (PMC13505945; doi:10.1371/journal.pone.0357041)
Supplement: Supplementary file 2 [file pone.0357041.s007.docx]

**RESPONSE TO THE REVIEWERS**

Thank you very much for such thorough and scholarly feedback on this manuscript. The reviewer’s comments are invaluable in improving our manuscript. We present our responses to the comments made by the reviewer, as shown below:

**Editor**

| **Comments** | **Response** |
| --- | --- |
| The reported interval of 2.80 ± 3.05 years appears to refer to the time between the participant’s last interview and death, rather than between death and the proxy interview. Therefore, it does not measure recall delay. Please report the correct interval, if available, or state that it cannot be calculated. | We agree, and thank the editor for pointing this out. The 2.80 ± 3.05-year interval reflects the time between the participant's last core interview and death, not the interval between death and the proxy interview. As the exact date of each proxy interview was not recorded, the true recall interval cannot be calculated. We have revised the Limitations section accordingly, clarifying what the reported interval represents and noting that recall bias remains a concern in principle, given that proxy interviews necessarily occur after death. (Manuscript, Limitations section, page 24) |
| Please also avoid stating that symptoms were inadequately treated or were early predictors of death, as these aspects were not evaluated. | We thank the editor for this comment. We have removed the statement that symptoms "remain inadequately recognized or treated as death approaches," as treatment adequacy was not evaluated in this study. We have also removed the description of loss of appetite as a "potential indicator of imminent death" and revised the description of loss of appetite, severe fatigue, and depression as "early indicators of approaching death," since their prognostic value for predicting death was not assessed. The relevant sentences now describe these symptoms only as markers of illness progression and as warranting priority attention in end-of-life symptom assessment, without implying a predictive role. (Manuscript, Discussion, page 20) |
| Explanations concerning delayed palliative care and NSAID use should be presented as possible hypotheses. | We thank the editor for this comment. We have revised both explanations to clearly present them as speculative hypotheses rather than established interpretations, as neither was directly tested in this study. (Manuscript, Discussion, page 20) |
| Please briefly address multiple testing across the 11 models and, | As suggested, we have briefly addressed this issue in the Statistical Analysis section and noted it as a limitation. (Manuscript, Statistical Analysis, page 11; Limitations, page 24) |
| if possible, perform a sensitivity analysis excluding “don’t know” responses. | As suggested, we conducted a sensitivity analysis excluding "don't know" responses for each symptom outcome (excluded n=5–16 per symptom), using the same models as the primary analysis. The method is described in the Statistical Analysis section, and the results—largely consistent with the primary analysis in direction and significance, with minor discrepancies limited to borderline cases—are reported in the Results section (Supplementary Table S4). (Manuscript, Statistical Analysis, page 11; Results, page 17) |
