# Supplementary material for: Prevalence and influencing factors of end-of-life symptoms among hospital decedents in South Korea: A cross-sectional secondary analysis
Source: PLoS One. 2026 Aug 25;21(8):e0357041. doi: 10.1371/journal.pone.0357041 (PMC13505945; doi:10.1371/journal.pone.0357041)
Supplement: Peer review history

# Peer review: Decision Letter 0

**DOI:** 10.1371/journal.pone.0357041.r001

**Authors:** Francesco Curcio

10 Apr 2026

Dear Dr. Eun Young Choi ,

Thank you for submitting your manuscript to PLOS ONE. After careful consideration, we feel that it has merit but does not fully meet PLOS ONE’s publication criteria as it currently stands. Therefore, we invite you to submit a revised version of the manuscript that addresses the points raised during the review process.

==============================

The reviewers acknowledge the value of the dataset and the relevance of the topic, but request substantial revision. The main concerns relate to limited analytical depth (overly descriptive approach, lack of alternative models and subgroup analyses), and important methodological issues (proxy-reported data, dichotomous symptom measures, potential misclassification and residual confounding). In addition, greater transparency is needed in the methods (definitions, handling of missing data, model specifications), as well as improvements in data presentation and interpretation (e.g., counterintuitive ORs, insufficient discussion of the hospital setting). Structural aspects (abstract, explicit research questions, clarification of hospital vs. hospice care) and a stronger, more critical discussion and limitations section are also required.

==============================

Please submit your revised manuscript by May 25 2026 11:59PM. If you will need more time than this to complete your revisions, please reply to this message or contact the journal office at plosone@plos.org. When you're ready to submit your revision, log on to https://www.editorialmanager.com/pone/ and select the 'Submissions Needing Revision' folder to locate your manuscript file.

- A letter that responds to each point raised by the academic editor and reviewer(s). You should upload this letter as a separate file labeled 'Response to Reviewers'.
- A marked-up copy of your manuscript that highlights changes made to the original version. You should upload this as a separate file labeled 'Revised Manuscript with Track Changes'.
- An unmarked version of your revised paper without tracked changes. You should upload this as a separate file labeled 'Manuscript'.

If you would like to make changes to your financial disclosure, please include your updated statement in your cover letter. Guidelines for resubmitting your figure files are available below the reviewer comments at the end of this letter.

If applicable, we recommend that you deposit your laboratory protocols in protocols.io to enhance the reproducibility of your results. Protocols.io assigns your protocol its own identifier (DOI) so that it can be cited independently in the future. For instructions see: https://journals.plos.org/plosone/s/submission-guidelines#loc-laboratory-protocols. Additionally, PLOS ONE offers an option for publishing peer-reviewed Lab Protocol articles, which describe protocols hosted on protocols.io. Read more information on sharing protocols at https://plos.org/protocols?utm_medium=editorial-email&utm_source=authorletters&utm_campaign=protocols.

As the corresponding author, your ORCID iD is verified in the submission system and will appear in the published article. PLOS supports the use of ORCID, and we encourage all coauthors to register for an ORCID iD and use it as well. Please encourage your coauthors to verify their ORCID iD within the submission system before final acceptance, as unverified ORCID iDs will not appear in the published article. *Only*  the individual author can complete the verification step; PLOS staff *cannot*  verify ORCID iDs on behalf of authors.

We look forward to receiving your revised manuscript.

Kind regards,

Francesco Curcio, M.D., Ph.D.

Academic Editor

PLOS One

Journal requirements:

When submitting your revision, we need you to address these additional requirements.

1. Please ensure that your manuscript meets PLOS ONE's style requirements, including those for file naming. The PLOS ONE style templates can be found at

https://journals.plos.org/plosone/s/file?id=wjVg/PLOSOne_formatting_sample_main_body.pdf and

https://journals.plos.org/plosone/s/file?id=ba62/PLOSOne_formatting_sample_title_authors_affiliations.pdf

If the reviewer comments include a recommendation to cite specific previously published works, please review and evaluate these publications to determine whether they are relevant and should be cited. There is no requirement to cite these works unless the editor has indicated otherwise.

Additional Editor Comments:

The reviewers acknowledge the value of the dataset and the relevance of the topic, but request substantial revision. The main concerns relate to limited analytical depth (overly descriptive approach, lack of alternative models and subgroup analyses), and important methodological issues (proxy-reported data, dichotomous symptom measures, potential misclassification and residual confounding). In addition, greater transparency is needed in the methods (definitions, handling of missing data, model specifications), as well as improvements in data presentation and interpretation (e.g., counterintuitive ORs, insufficient discussion of the hospital setting). Structural aspects (abstract, explicit research questions, clarification of hospital vs. hospice care) and a stronger, more critical discussion and limitations section are also required.

[Note: HTML markup is below. Please do not edit.]

Reviewers' comments:

Reviewer's Responses to Questions

**Comments to the Author**

1. Is the manuscript technically sound, and do the data support the conclusions?

Reviewer #1: Yes

Reviewer #2: Yes

**********

2. Has the statistical analysis been performed appropriately and rigorously?

Reviewer #1: Yes

Reviewer #2: Yes

**********

3. Have the authors made all data underlying the findings in their manuscript fully available?

The PLOS Data policy

Reviewer #1: Yes

Reviewer #2: Yes

**********

4. Is the manuscript presented in an intelligible fashion and written in standard English?

Reviewer #1: Yes

Reviewer #2: Yes

**********

Reviewer #1: Reviewer comments to

Prevalence and influencing factors of symptoms in the last month of life 2 among patients who died in hospitals

The authors have analyzed over a 1000 symptom questionnaires of patients dying in a hospital, collected by proxy response after death. They found a high prevalence of symptoms. The proportion of people dying in a hospital is very high in Korea.

It is a well written article, and the authors provide thorough and right implications for improving care.

Although I praise the authors for this work and their effort I doubt whether the new finding are newsworthy for an international public, also given the design with proxy responses.

The major flaw is that the collected data are not only retrospective but also not provided by patients self but only by an interview with the next of kin after death of patients. In addition, the answers are dichotomous and not scaling, therefore yes or no, leading to overestimation perhaps. This all has a high risk of bias. This is however unavoidable given the database as the authors admit.

It is not known whether or not these symptoms were addressed during the hospital stay. To me it is surprising that so many symptoms exist in such a controlled environment with professional health care givers in a hospital. Could the authors comment on that?

Specific comments: page 9. The authors mention hospitalization but also hospice ward. Please explain, is there a difference according to the authors, is a hospice admission the same as an hospital admission or is a hospice a ward of a normal hospital or are there combinations?

Reviewer #2: The manuscript addresses an important topic: the prevalence and influencing factors of end‑of‑life symptoms among hospitalized patients in South Korea. It contributes useful data drawn from the nationally representative Korean Longitudinal Study of Aging (KLoSA), covering a long period (2006–2020). The findings could provide valuable evidence for palliative‑care policy and clinical practice.

However, substantial revision is required before the paper can be considered for publication. Key concerns involve conceptual clarity, methodological specificity, data presentation, and interpretation of results. The paper is currently too descriptive and lacks sufficient analytical depth to support its conclusions.

1. Abstract and Introduction

Major points

The abstract is overly long and includes background details better suited for the introduction. Condense it to 250–300 words, emphasizing the objective, key methods, major results, and main implications.

The background repeats several ideas (functional decline, quality of dying, caregiver burden). Streamline to highlight the knowledge gap—namely, the lack of studies examining symptom prevalence and associated factors among hospital decedents in Korea.

Clarify the research questions or hypotheses explicitly at the end of the introduction. For example:

“This study aimed to (1) determine the prevalence of end‑of‑life symptoms experienced in the last month of life among hospitalized decedents in South Korea, and (2) identify demographic, health, and care-related factors associated with these symptoms.”

2. Methods

Study design and population

The use of cross-sectional design on KLoSA exit interview data should be described more precisely. Indicate whether this secondary analysis included all proxy respondents or only those with complete symptom data.

Clarify how “death in hospital” was defined—e.g., based on proxy report or KLoSA administrative linkage—and discuss potential misclassification.

The exclusion process shown in Fig. 1 must specify how many participants were removed for missing covariates or weighting data.

Variables

List each symptom exactly as coded in KLoSA, specifying the response categories and how missing or “don’t know” responses were handled.

The ADL and IADL scores should include the scoring range and the threshold, if any, for “dependency.”

Clarify how the variable duration of illness before death was re‑categorized for regression analysis—currently presented inconsistently (lines 161–164 vs. 263 table).

Statistical analysis

More justification is needed for using 11 separate logistic regressions rather than a multivariate or count‑based model (e.g., Poisson or negative binomial) for symptom number.

Indicate which confounders were included in each model and whether multicollinearity was tested.

Specify how sampling weights were applied in SPSS (complex sample module, strata, cluster).

Include the total number of observations used in each regression model to assess sample attrition.

3. Results

Presentation

Tables 1–3 are informative but dense. Simplify by grouping variables thematically or relocating supplementary details to an appendix.

Provide unweighted n and weighted % or mean consistently.

Consider a separate figure with stacked bars showing the top five symptoms to visualize overall burden more clearly than the current textual description.

Interpretation

The mean number of 4.76 ± 0.12 symptoms is reported, but no measure of distribution or comparison by subgroups (e.g., age, sex, illness duration) is offered. Include analyses showing how symptom burden varies across key characteristics.

Some odds ratios appear counterintuitive (e.g., arthritis inversely associated with loss of appetite and depression). Discuss potential confounding, measurement issues, or clinical explanations.

The statement that dyspnea was not a “major symptom” contradicts its 48.8 % prevalence—clarify your comparison criteria.

4. Discussion

Depth and structure

Currently too long and largely descriptive. Organize around (1) main findings, (2) comparison with prior studies, (3) possible mechanisms, and (4) clinical implications.

Avoid repeating numerical results; interpret instead what these indicate about end‑of‑life care quality or system gaps.

The discussion should address why the duration of illness, ADL/IADL dependence, and pain are so strongly linked with most symptoms. Are these causal, or proxies for disease severity?

Missed areas

Include a more explicit reflection on the hospital setting—why symptom management may differ from home or hospice deaths.

Expand on policy implications in Korea’s current hospice‑care context rather than only recommending “multidisciplinary care.”

See and discuss:

Depression:

1) Cacciatore F et al.. Effect of Sacubitril-Valsartan in reducing depression in patients with advanced heart failure. J Affect Disord. 2020 Jul 1;272:132-137.

Frailty and heart failure:

2) Testa G et al. Multidimensional frailty evaluation in elderly outpatients with chronic heart failure: A prospective study. Eur J Prev Cardiol. 2019 Jul;26(10):1115-1117.

Catabolic syndrome:

3 Curcio F et al. The reverse metabolic syndrome in the elderly: Is it a "catabolic" syndrome? Aging Clin Exp Res. 2018 Jun;30(6):547-554.

5. Limitations

The limitations section is appropriate but should be more analytic:

Quantify the potential recall bias (delay between death and exit interview).

Discuss non‑response bias and whether excluded cases differed systematically.

Note the limited ability to infer causality due to the cross‑sectional design.

Mention that proxy perceptions may systematically underestimate psychological symptoms (depression, emotion control).

6. Conclusion

Condense to one clear paragraph emphasizing what was discovered and how it advances practice.

Avoid generic statements like “this study provides foundational data.” Instead:

“Among hospitalized decedents aged ≥45 years in Korea, loss of appetite, severe fatigue, and depression were the most frequent end‑of‑life symptoms, each strongly associated with prolonged illness and higher dependency. Early recognition of these indicators may improve the quality of dying through targeted palliative interventions.”

**********

what does this mean?). If published, this will include your full peer review and any attached files.

If you choose “no”, your identity will remain anonymous but your review may still be made public.

**Do you want your identity to be public for this peer review?**  For information about this choice, including consent withdrawal, please see our Privacy Policy

Reviewer #1: No

Reviewer #2: **Yes:** Pasquale Abete

**********

[NOTE: If reviewer comments were submitted as an attachment file, they will be attached to this email and accessible via the submission site. Please log into your account, locate the manuscript record, and check for the action link "View Attachments". If this link does not appear, there are no attachment files.]

To ensure your figures meet our technical requirements, please review our figure guidelines: https://journals.plos.org/plosone/s/figures

You may also use PLOS’s free figure tool, NAAS, to help you prepare publication quality figures: https://journals.plos.org/plosone/s/figures#loc-tools-for-figure-preparation.

NAAS will assess whether your figures meet our technical requirements by comparing each figure against our figure specifications.

# Author comment: Author response to Decision Letter 1

**DOI:** 10.1371/journal.pone.0357041.r002

17 May 2026

Thank you for the opportunity to revise our manuscript. We have carefully addressed all comments from the Editor and both reviewers. Our detailed point-by-point responses are provided in the attached "Response to Reviewers" document.

Attachment Submitted filename: Symptoms_Response to Reviewers_R1_260516.docx

# Peer review: Decision Letter 1

**DOI:** 10.1371/journal.pone.0357041.r003

**Authors:** Francesco Curcio

20 Jul 2026

Dear Dr. Choi,

Thank you for submitting your manuscript to PLOS ONE. After careful consideration, we feel that it has merit but does not fully meet PLOS ONE’s publication criteria as it currently stands. Therefore, we invite you to submit a revised version of the manuscript that addresses the points raised during the review process.

==============================

The manuscript has substantially improved, and the reviewers’ comments have generally been addressed. However, a few points should be corrected before acceptance. The reported interval of 2.80 ± 3.05 years appears to refer to the time between the participant’s last interview and death, rather than between death and the proxy interview. Therefore, it does not measure recall delay. Please report the correct interval, if available, or state that it cannot be calculated. Please also avoid stating that symptoms were inadequately treated or were early predictors of death, as these aspects were not evaluated. Explanations concerning delayed palliative care and NSAID use should be presented as possible hypotheses. Finally, please briefly address multiple testing across the 11 models and, if possible, perform a sensitivity analysis excluding “don’t know” responses. After these corrections, the manuscript may be suitable for acceptance.

==============================

Please submit your revised manuscript by Sep 03 2026 11:59PM. If you will need more time than this to complete your revisions, please reply to this message or contact the journal office at plosone@plos.org. When you're ready to submit your revision, log on to https://www.editorialmanager.com/pone/ and select the 'Submissions Needing Revision' folder to locate your manuscript file.

- A letter that responds to each point raised by the academic editor and reviewer(s). You should upload this letter as a separate file labeled 'Response to Reviewers'.
- A marked-up copy of your manuscript that highlights changes made to the original version. You should upload this as a separate file labeled 'Revised Manuscript with Track Changes'.
- An unmarked version of your revised paper without tracked changes. You should upload this as a separate file labeled 'Manuscript'.

If you would like to make changes to your financial disclosure, please include your updated statement in your cover letter. Guidelines for resubmitting your figure files are available below the reviewer comments at the end of this letter.

If applicable, we recommend that you deposit your laboratory protocols in protocols.io to enhance the reproducibility of your results. Protocols.io assigns your protocol its own identifier (DOI) so that it can be cited independently in the future. For instructions see: https://journals.plos.org/plosone/s/submission-guidelines#loc-laboratory-protocols. Additionally, PLOS ONE offers an option for publishing peer-reviewed Lab Protocol articles, which describe protocols hosted on protocols.io. Read more information on sharing protocols at https://plos.org/protocols?utm_medium=editorial-email&utm_source=authorletters&utm_campaign=protocols.

As the corresponding author, your ORCID iD is verified in the submission system and will appear in the published article. PLOS supports the use of ORCID, and we encourage all coauthors to register for an ORCID iD and use it as well. Please encourage your coauthors to verify their ORCID iD within the submission system before final acceptance, as unverified ORCID iDs will not appear in the published article. *Only*  the individual author can complete the verification step; PLOS staff *cannot*  verify ORCID iDs on behalf of authors.

We look forward to receiving your revised manuscript.

Kind regards,

Francesco Curcio, M.D., Ph.D.

Academic Editor

PLOS One

**Journal Requirements:**

1. If the reviewer comments include a recommendation to cite specific previously published works, please review and evaluate these publications to determine whether they are relevant and should be cited. There is no requirement to cite these works unless the editor has indicated otherwise.

2. Please review your reference list to ensure that it is complete and correct. If you have cited papers that have been retracted, please include the rationale for doing so in the manuscript text, or remove these references and replace them with relevant current references. Any changes to the reference list should be mentioned in the rebuttal letter that accompanies your revised manuscript. If you need to cite a retracted article, indicate the article’s retracted status in the References list and also include a citation and full reference for the retraction notice.

**Additional Editor Comments:**

The manuscript has substantially improved, and the reviewers’ comments have generally been addressed. However, a few points should be corrected before acceptance. The reported interval of 2.80 ± 3.05 years appears to refer to the time between the participant’s last interview and death, rather than between death and the proxy interview. Therefore, it does not measure recall delay. Please report the correct interval, if available, or state that it cannot be calculated. Please also avoid stating that symptoms were inadequately treated or were early predictors of death, as these aspects were not evaluated. Explanations concerning delayed palliative care and NSAID use should be presented as possible hypotheses. Finally, please briefly address multiple testing across the 11 models and, if possible, perform a sensitivity analysis excluding “don’t know” responses. After these corrections, the manuscript may be suitable for acceptance.

[Note: HTML markup is below. Please do not edit.]

Reviewers' comments:

Reviewer's Responses to Questions

**Comments to the Author**

Reviewer #1: All comments have been addressed

Reviewer #2: All comments have been addressed

**********

2. Is the manuscript technically sound, and do the data support the conclusions?

Reviewer #1: Yes

Reviewer #2: Yes

**********

3. Has the statistical analysis been performed appropriately and rigorously?

Reviewer #1: Yes

Reviewer #2: Yes

**********

4. Have the authors made all data underlying the findings in their manuscript fully available?

The PLOS Data policy

Reviewer #1: Yes

Reviewer #2: Yes

**********

5. Is the manuscript presented in an intelligible fashion and written in standard English?

Reviewer #1: Yes

Reviewer #2: Yes

**********

**Reviewer #1:** my previous concerns and comments have been addresed and integrated into the revised version

**Reviewer #2:** Dear Editor,

the manuscript is really improved. All questions have been answered and the manuscript merits to be published in PONE.

Sincerely,

**********

what does this mean?). If published, this will include your full peer review and any attached files.

If you choose “no”, your identity will remain anonymous but your review may still be made public.

**Do you want your identity to be public for this peer review?**  For information about this choice, including consent withdrawal, please see our Privacy Policy

Reviewer #1: **Yes:** dr wk de jong

Reviewer #2: No

**********

[NOTE: If reviewer comments were submitted as an attachment file, they will be attached to this email and accessible via the submission site. Please log into your account, locate the manuscript record, and check for the action link "View Attachments". If this link does not appear, there are no attachment files.]

To ensure your figures meet our technical requirements, please review our figure guidelines: https://journals.plos.org/plosone/s/figures

You may also use PLOS’s free figure tool, NAAS, to help you prepare publication quality figures: https://journals.plos.org/plosone/s/figures#loc-tools-for-figure-preparation.

NAAS will assess whether your figures meet our technical requirements by comparing each figure against our figure specifications.

# Author comment: Author response to Decision Letter 2

**DOI:** 10.1371/journal.pone.0357041.r004

2 Aug 2026

Please see our point-by-point responses to the Academic Editor's comments in the attached "Response to Reviewers" document.

Attachment Submitted filename: Symptoms_Response to Reviewers_R2_260723.docx

# Editor report: Decision Letter 2

**DOI:** 10.1371/journal.pone.0357041.r005

**Authors:** Francesco Curcio

11 Aug 2026

Prevalence and influencing factors of end-of-life symptoms among hospital decedents in South Korea: a cross-sectional secondary analysis

PONE-D-26-02493R2

Dear Dr. Eun Young Choi ,

We’re pleased to inform you that your manuscript has been judged scientifically suitable for publication and will be formally accepted for publication once it meets all outstanding technical requirements.

Within one week, you’ll receive an e-mail detailing the required amendments. When these have been addressed, you’ll receive a formal acceptance letter and your manuscript will be scheduled for publication.

An invoice will be generated when your article is formally accepted. Please note, if your institution has a publishing partnership with PLOS and your article meets the relevant criteria, all or part of your publication costs will be covered. Please make sure your user information is up-to-date by logging into Editorial Manager at Editorial Manager® and clicking the ‘Update My Information' link at the top of the page. For questions related to billing, please contact billing support.

If your institution or institutions have a press office, please notify them about your upcoming paper to help maximize its impact. If they’ll be preparing press materials, please inform our press team as soon as possible -- no later than 48 hours after receiving the formal acceptance. Your manuscript will remain under strict press embargo until 2 pm Eastern Time on the date of publication. For more information, please contact onepress@plos.org.

Kind regards,

Francesco Curcio, M.D., Ph.D.

Academic Editor

PLOS One

Additional Editor Comments (optional):

The manuscript has been substantially improved, and the authors have adequately addressed the issues raised. In its current form, the manuscript merits publication in PLOS ONE.

Reviewers' comments:

# Editor report: Acceptance letter

**DOI:** 10.1371/journal.pone.0357041.r006

**Authors:** Francesco Curcio

PONE-D-26-02493R2

PLOS One

Dear Dr. Choi,

I'm pleased to inform you that your manuscript has been deemed suitable for publication in PLOS One. Congratulations! Your manuscript is now being handed over to our production team.

At this stage, our production department will prepare your paper for publication. This includes ensuring the following:

* All references, tables, and figures are properly cited

* All relevant supporting information is included in the manuscript submission,

* There are no issues that prevent the paper from being properly typeset

You will receive further instructions from the production team, including instructions on how to review your proof when it is ready. Please keep in mind that we are working through a large volume of accepted articles, so please give us a few days to review your paper and let you know the next and final steps.

Lastly, if your institution or institutions have a press office, please let them know about your upcoming paper now to help maximize its impact. If they'll be preparing press materials, please inform our press team within the next 48 hours. Your manuscript will remain under strict press embargo until 2 pm Eastern Time on the date of publication. For more information, please contact onepress@plos.org.

You will receive an invoice from PLOS for your publication fee after your manuscript has reached the completed accept phase. If you receive an email requesting payment before acceptance or for any other service, this may be a phishing scheme. Learn how to identify phishing emails and protect your accounts at https://explore.plos.org/phishing.

If we can help with anything else, please email us at customercare@plos.org.

Thank you for submitting your work to PLOS One and supporting open access.

Kind regards,

PLOS ONE Editorial Office Staff

on behalf of

Dr. Francesco Curcio

Academic Editor

PLOS One
